# Adverse effect of age on pancreatic islet morphology, islet cell turnover and lineage-labelled hormone expression in mice

**DOI:** 10.1007/s40618-026-02884-6

**Published:** 2026-04-16

**Authors:** V. Dubey, N. Tanday, A. Sridhar, A. I. Tarasov, P. R. Flatt, R. C. Moffett, N. Irwin

**Affiliations:** Centre for Diabetes, School of Biomedical Sciences, Ulster University, Cromore Road, Coleraine, Northern Ireland BT52 1SA UK

**Keywords:** Ageing, Beta-cell mass, Hormone expression, Islet remodelling, Lineage tracing

## Abstract

**Aims:**

Ageing leads to a gradual decline in the structure and function of many bodily organs, including the endocrine pancreas. The current study examines the impact of ageing on pancreatic islet morphology, and especially the role of islet cell plasticity in this process.

**Methods:**

Transgenic *Ins1*^*Cre/*+^*;Rosa26-eYFP* mice, with islet beta-cell tracing capabilities, were employed at 12 and 52 weeks of age, and islet morphology, islet cell turnover as well as changes in beta-cell identity and plasticity assessed.

**Results:**

Ageing was associated with elevated body weight and circulating glucose levels. There was also substantial remodelling of pancreatic islet morphology in 52-week-old mice, that included a notable decrease of islet number as well as overall islet, alpha- and beta-cell areas. This was associated with diminished beta-cell proliferation and survival, but interestingly alpha-cell proliferation was increased in older mice, as was the number of islet cells positive for both insulin and glucagon. There was increased loss of insulin expression in original GFP labelled islet cells in older *Ins1*^*Cre/*+^*;Rosa26-eYFP* mice complemented by augmented glucagon and GFP co-positive cell numbers. Observations in *Glu*^*CreERT2*^*;ROSA26-eYFP* transgenic mice, with alpha-cell tracing technologies, demonstrated reduced numbers of lineage-labelled alpha-cells co-expressing insulin. Furthermore, the ability of pancreatic ductal cells to assume an islet beta-cell phenotype was impaired in older *Ins1*^*Cre/*+^*;Rosa26-eYFP* mice.

**Conclusion:**

These findings demonstrate an age-related deterioration of pancreatic islet morphology, linked in part to altered patterns of lineage-labelled hormone expression alongside changes in islet cell turnover.

**Supplementary Information:**

The online version contains supplementary material available at10.1007/s40618-026-02884-6.

## Introduction 

Ageing is a major risk factor for impaired glucose tolerance and type 2 diabetes mellitus (T2DM) [1]. Although ageing-related insulin resistance is well documented [2], the mechanisms driving pancreatic islet dysfunction in this context remain incompletely understood. Current evidence suggests that sustained peripheral insulin resistance initially provokes compensatory beta-cell expansion and enhanced insulin secretion [3]. However, over time this adaptation often fails, leading to functional decline and loss of beta-cell identity [4].

Conventionally, loss of functional beta-cell mass has been attributed to induction of cellular apoptosis [2], consistent with the inherently low antioxidant defences of beta-cells [5]. Yet, the majority of studies report no obvious change in beta-cell apoptosis rates between young and old individuals [6], prompting the search for alternative mechanisms. In this respect, attention has turned to loss of adult beta-cell identity and related beta-cell dedifferentiation, characterised by a downregulation in specific transcription factors essential for maintaining mature beta-cell phenotype [7]. When viewed in the context that DNA instability is a classic hallmark of ageing [8], detrimental alterations of islet cell lineage may represent an important underlying mechanism behind diminished pancreatic islet function in ageing [9]. In harmony with this, recent studies highlight negative changes of pancreatic islet beta-cell plasticity to play a significant role in the pathogenesis of T2DM [10]. Specifically, dedifferentiation of mature beta-cells, through downregulation of key genes including Pdx1, MafA and FoxO1, results in beta-cells that are unable to sense glucose levels and/or secrete adequate amounts of insulin, a well-recognised aspect in the progression of T2DM [10]. Similarly, transdifferentiation of mature beta-cells towards a cell lineage type more akin with alpha- or delta-cells represents a potentially reversible aspect of decreased beta-cell function in T2DM [10]. Despite these advances, a critical knowledge gap remains in relation to the extent physiological ageing alters the balance between maintenance of beta-cell identity, transdifferentiation of new beta-cells from other endocrine or ductal cells as well as rates of beta-cell dedifferentiation. Addressing this matter is essential to understanding how ageing predisposes to T2DM and in identifying potential targets for disease intervention.

Therefore, in the present study, we have used transgenic mice with beta-cell lineage tracing capabilities, namely *Ins1*^*Cre/*+^*;Rosa26-eYFP* mice [11, 12], to investigate normal physiological age-induced changes in pancreatic islet morphology, islet cell turnover rates and lineage-labelled hormone expression patterns. Employing transgenic mice at 12 and 52 weeks of age, we hypothesised that disturbed pancreatic islet function in ageing would be linked to adverse changes in islet cell plasticity leading to loss of beta-cell identity. Initial parallel observations were made in similarly aged transgenic *Glu*^*CreERT2*^*;ROSA26-eYFP* mice, with islet alpha-cell lineage tracing technology. We hypothesised that impaired glucose tolerance classically observed in older animals would be associated with reduced beta-cell proliferation and survival, as well as negative effects on lineage-labelling of islet cells that would tend to promote a functional beta-cell phenotype.

## Materials and methods

### Animals

*Ins1*^*Cre/*+^*;Rosa26-eYFP* male mice were bred in house within the Biomedical and Behavioural Research Unit at Ulster University, Coleraine, UK and used at either 12 or 52 weeks of age. This age range was chosen based on previous studies in our laboratory confirming metabolic impairment in mice at approximately 52 weeks of age [13, 14]. The origin and characteristics of these transgenic mice, with Cre recombinase enzyme expression under the control of the Insulin 1 (*Ins1*) promoter, specifically targeting pancreatic beta-cells, have been described previously [15, 16]. Studies were also conducted in male *Glu*^*CreERT2*^*;ROSA26-eYFP* transgenic mice a the Cre recombinase enzyme under the control of the glucagon promoter in pancreatic alpha-cells, as described previously [17]. All mice were housed in a temperature-controlled environment (22 ± 2 °C) on a 12-h light/dark cycle. They were maintained on standard chow (10% fat content, Trouw Nutrition, Norwich, UK) and normal drinking water ad libitum. All experiments were approved by Ulster University Animal Ethics Review Committee, conducted in accordance with the UK Animals (Scientific Procedures) Act 1986, and reported in line with the ARRIVE (Animal Research: Reporting of In Vivo Experiments) guidelines.

### Immunohistochemistry

Mice were euthanised at 12- or 52-weeks by lethal inhalation of CO_2_ followed by cervical dislocation, with body weight and blood glucose determined just prior to this, and insulin concentrations determined from terminally collected plasma samples. Blood glucose was measured using a hand-held Ascencia Contour blood glucose meter (Bayer Healthcare, Newbury, Berkshire, UK) and plasma insulin assessed by an in-house insulin radioimmunoassay [11]. Immediately following euthanasia, pancreatic tissue was excised from 12- or 52-week-old mice and fixed in 4% paraformaldehyde in PBS (pH 7.4) for 48 h at room temperature, followed by dehydration through a graded ethanol series (50%, 60%, 70%, 80%, 90%, 95% and 100%; 1 h each) and clearing in xylene (2 × 30 min). Samples were then embedded in paraffin wax using a tissue processor and serial sections (5 µm thickness) prepared, as described previously [16]. To confirm reliability, tissue orientation was consistently maintained with the long axis orientated parallel to the blade of the microtome and morphometric analysis conducted on every tenth section throughout each pancreas. Antigen retrieval was achieved by immersion in heated 10 mM citrate buffer (90 °C, pH6) followed by blocking in 4% bovine serum albumin (BSA). Primary antibodies, including insulin, glucagon, GFP, Ki-67 and CK-19 were then added (18 h incubation at 37 °C) followed by appropriate secondary antibodies (1 h incubation at 37 °C), employing dilution factors listed in supplementary Table 1. Notably, our GFP antibody is reactive against all variants of *Aequorea Victoria* GFP, including YFP. Lastly, slides were exposed to DAPI to identify nuclei before washing in PBS and mounting with glass coverslips. For assessing apoptosis, commercially available TUNEL staining (Roche Diagnostics, UK) was carried out following the manufacturer’s guidance. Stained slides were imaged on an Olympus BX-51 fluorescent microscope fitted with DAPI (350 nm), TRITC (594 nm) and FITC (488 nm) filters.

### Image analysis

Cell^F^ imaging software (Olympus Soft Imaging Solutions) was employed to assess islet morphology as well as cellular proliferation, apoptosis, changes in lineage-labelled hormone expression and neogenesis, with blinded analysis of all images. Slides stained for insulin/glucagon were used to assess basic islet morphology and quantified on ImageJ software using a “closed polygon” tool, being expressed as total islet, beta- and alpha-cell areas [18]. Briefly, the closed polygon tool allows enclosure of a selected segment within an image for measuring area in μm^2^. In doing so, total islet area can be measured by considering the combined aggregate of all insulin- and glucagon-positively stained cells, beta-cell area as all insulin-positive cells, alpha-cell area as all glucagon-positive cells and dual hormone positive cells as those cells expressing both insulin and glucagon. Slides stained with TUNEL or Ki-67 were used to assess apoptosis and proliferation, respectively, in either insulin or glucagon positive cells, and data expressed as a percentage of total cells analysed. Similarly, ductal cell hormone expression was quantified by assessing the percentage of CK-19 positively stained pancreatic ductal cells co-expressing insulin. For islet endocrine lineage analysis, it is important to note that in transgenic *Ins1*^*Cre/*+^*;Rosa26-eYFP* mice, GFP-positive cells are always of initial beta-cell lineage. Thus, islet cells expressing both insulin and GFP are considered original beta-cells, whereas cells positive for GFP but lacking insulin (Insulin^−ve^, GFP^+ve^), cells expressing insulin without GFP (Insulin^+ve^, GFP^−ve^) and cells positive for both glucagon and GFP (Glucagon^+ve^, GFP^+ve^) have altered lineage-labelled hormone expression. In *Glu*^*CreERT2*^*;ROSA26-eYFP* transgenic mice, cells expressing both glucagon and GFP were considered original alpha-cells whereas cells staining positive for insulin and GFP (Insulin^−ve^, GFP^+ve^) exhibit altered lineage-labelled hormone expression. For each parameter assessed, > 60 islets were analysed per treatment group (n = 5–7 animals per group), and morphometric analysis performed on each individual mouse (at least 10 islets per mouse) with every tenth section used to ensure different islets were studied, as previously described [19].

### Statistical analyses

**Fig. 1:**
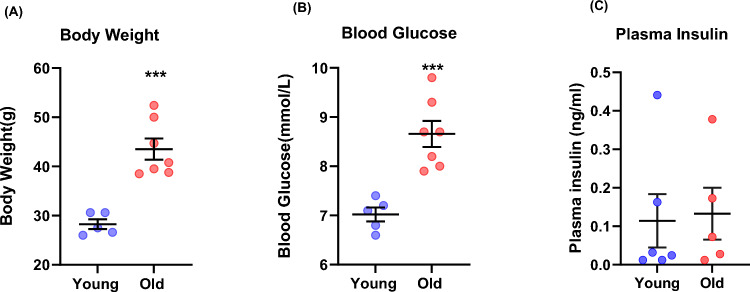
(**A**) Body weight, (**B**) blood glucose and (**C**) plasma insulin in 12- and 52-week-old *Ins1*^*Cre/*+^*;Rosa26-eYFP* mice. Values are mean ± SEM (n = 5–7 animals per group) with data points representing individual animals. **p < 0.01 compared to 12-week-old control mice using Mann–Whitney U-test

**Fig. 2:**
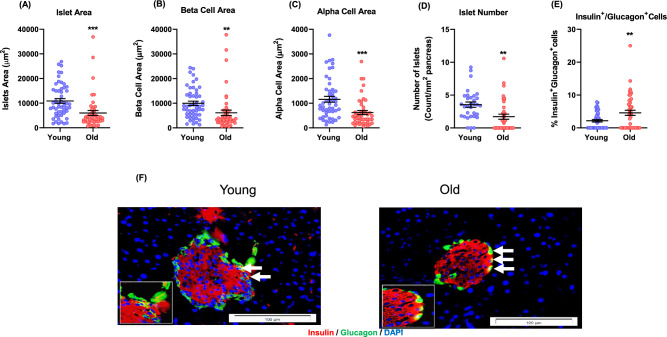
Pancreatic islet morphology in 12- and 52-week-old *Ins1*^*Cre/*+^*;Rosa26-eYFP* mice. Immunohistochemistry was conducted to assess (**A**) islet area, (**B**) beta-cell area, (**C**) alpha-cell area, (**D**) islet number as well as (**E**) double hormone positive islet cells. (**F**) Representative islet images from 12- and 52-week-old mice showing insulin (red), glucagon (green) and DAPI (blue), with arrows depicting double hormone positive cells. Values are mean ± SEM (60 islets from n = 5–7 animals per group; at least 10 islets per mouse) with data points representing individual islets. **p < 0.01 and ***p < 0.001 compared to 12-week-old control mice using independent-samples t-test

**Fig. 3:**
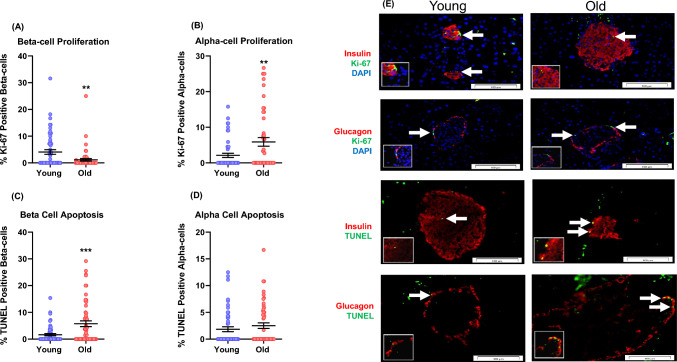
Islet beta-cell and alpha-cell proliferation and apoptosis in 12- and 52-week-old *Ins1*^*Cre/*+^*;Rosa26-eYFP* mice. Immunohistochemistry was conducted to assess (**A**) beta-cell proliferation, (**B**) alpha-cell proliferation, (**C**) beta-cell apoptosis and (**D**) alpha-cell apoptosis. (**E**) Representative images showing DAPI (blue), Ki-67 and TUNEL (green) staining, with arrows highlighting positive co-staining alongside insulin or glucagon (red), as appropriate. Values are mean ± SEM (60 islets from n = 5–7 animals per group; at least 10 islets per mouse) with data points representing individual islets. **p < 0.01 and ***p < 0.001 compared to 12-week-old control mice using independent-samples t-test

**Fig. 4:**
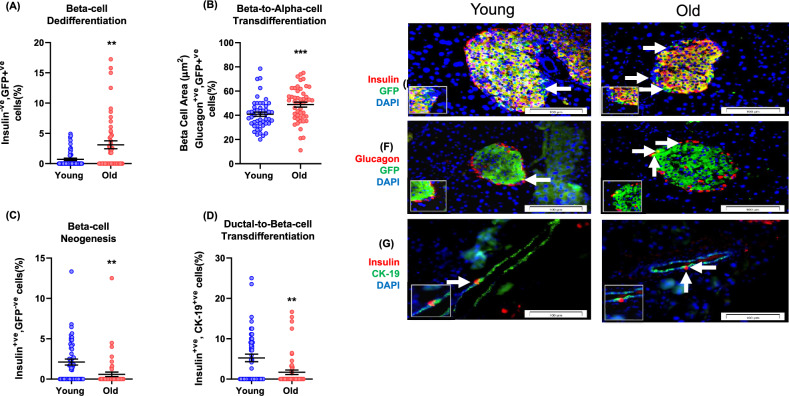
Beta-cell dedifferentiation, transdifferentiation and neogenesis in 12- and 52-week-old *Ins1*^*Cre/*+^*;Rosa26-eYFP* mice. Immunohistochemistry was conducted to assess (**A**) beta-cell dedifferentiation, (**B**) beta- to alpha-cell transdifferentiation, (**C**) beta-cell neogenesis and (**D**) ductal to beta-cell transdifferentiation. Representative images from each group showing (**E**) insulin (red), GFP (green) and DAPI (blue), (**F**) glucagon (red), GFP (green) and DAPI (blue) or (**G**) insulin (red), CK-19 (green) and DAPI (blue) with arrows highlighting positive co-staining, as appropriate. Values are mean ± SEM (60 islets from n = 5–7 animals per group; at least 10 islets per mouse) with data points representing individual islets. **p < 0.01 and ***p < 0.0001 compared to 12-week-old control mice using independent-samples t-test

**Fig. 5:**
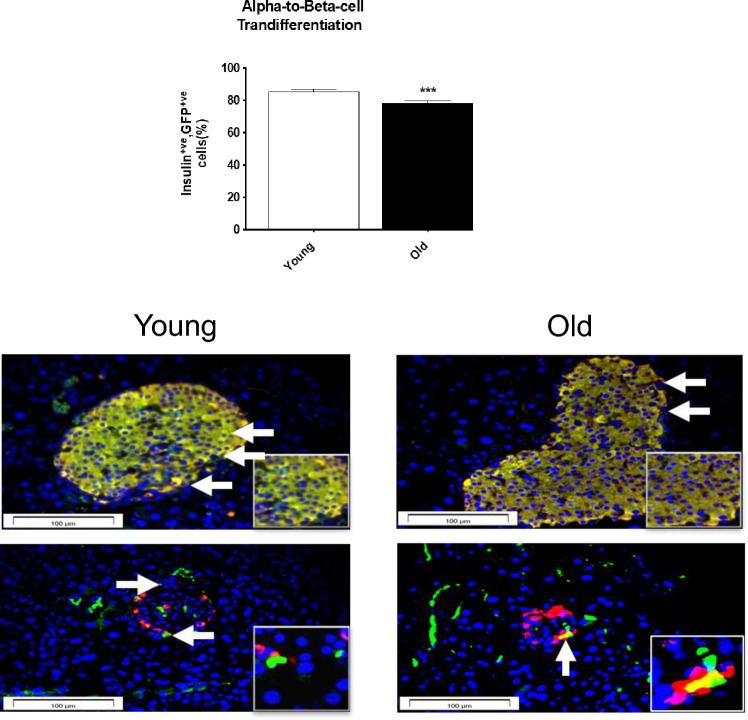
Alpha-cell transdifferentiation in 12- and 52-week-old *Glu*^*CreERT2*^*/Rosa26-eYFP* mice immunohistochemistry was conducted to assess alpha- to beta-cell transdifferentiation with a representative image from each group of mice showing glucagon (red), GFP (green) and DAPI (blue). Values are mean ± SEM (60 islets from n = 5–6 animals per group; at least 10 islets per mouse). ***p < 0.0001 compared to 12-week-old control mice

Statistical analysis was performed using R, whereas GraphPad PRISM (version 8) was utilised for exploratory data analysis. All analyses were performed on per-islet averages with at least 10 islets examined per mouse. All data is presented as the mean values ± S.E.M. Mann–Whitney U-test was used to compute the significance of differences between independent samples in Fig. 1; independent-samples t-test was employed for data presented in Figs. 2, 3, 4 and 5. Results were deemed significant once *P* < 0.05. Sample sizes for biological replicates are given in the figure legends.

## Results

### Ageing increases body weight and non-fasting glucose levels, with less effect on circulating insulin

Body weights and blood glucose levels (43.5 ± 2.1 *vs*. 28.2 ± 1.0 g and 8.6 ± 0.2 *vs*. 7.0 ± 0.1 mM; respectively) were significantly (*p* < 0.001) elevated in 52 week old *Ins1*^*Cre/*+^*;Rosa26-eYFP* transgenic mice when compared to 12 week old controls (Fig. 1A, B). Terminal plasma insulin concentrations were similar in both groups on mice (Fig. 1C).

### Ageing induces alterations in pancreatic islet morphology

Iselt, alpha- and beta-cell areas were decreased (*p* < 0.01–001) in 52 week old *Ins1*^*Cre/*+^*;Rosa26-eYFP* mice when compared to 12 week old controls (Fig. 2A-C), accompanied by fewer overall islet numbers (Fig. 2D). Overall, ageing reduced the size and number of pancreatic islets, with corresponding reductions in the area of insulin and glucagon positive cells (Fig. 2A-D). The number of dual-hormone positive islet cells co-expressing both insulin and glucagon were more than doubled (*p* < 0.01) in older *Ins1*^*Cre/*+^*;Rosa26-eYFP* mice (Fig. 2E). Figure 2F depicts images of islets stained for glucagon and insulin, with arrows highlighting dual hormone positive cells.

### Ageing alters rates of pancreatic islet cell turnover

There were reductions (*p* < 0.01) in beta-cell proliferation rates, alongside increased numbers of apoptotic beta-cells, in 52 week old *Ins1*^*Cre/*+^*;Rosa26-eYFP* mice when compared to 12 week old controls (Fig. 3A,C), suggesting ageing moderates beta-cell turnover. In addition, older mice also presented with elevated (*p* < 0.01) alpha-cell proliferation than 12 week old controls (Fig. 3B), but there was no obvious change in alpha-cell apoptosis rates between the two groups of mice (Fig. 3D). Characteristic images of alpha- and beta-cells co-stained for Ki-67 and TUNEL are shown in Fig. 3E.

### Ageing was associated with an increased proportion of lineage-labelled cells lacking insulin expression and reduced indicators of beta-cell neogenesis

An increased proportion of original GFP lineage-labelled cells lacking insulin expression was observed in older *Ins1*^*Cre/*+^*;Rosa26-eYFP* transgenic mice (*p* < 0.01), consistent with altered hormone marker profiles within their beta-cell population (Fig. 4A). In addition, the number of GFP-positive islet cells co-expressing glucagon was increased in older mice (*p* < 0.001), consistent with altered hormone expression in islet cells originating from a beta-cell lineage (Fig. 4B). Beta-cell neogenesis from an islet cell endocrine source was decreased (*p* < 0.01) in old mice (Fig. 4C). Furthermore, the proportion of CK-19 positive ductal cells co-expressing insulin was reduced (*p* < 0.01) in older mice when compared to younger controls (Fig. 4D). Representative images of islets co-stained for insulin and GFP (Fig. 4E), glucagon and GFP (Fig. 4F) as well as insulin and CK-19 (Fig. 4G) are also shown. Finally, initial pilot studies in *Glu*^*CreERT2*^*;ROSA26-eYFP* transgenic mice corroborated findings from *Ins1*^*Cre/*+^*;Rosa26-eYFP* mice, where ageing was associated with reduced (*p* < 0.001) numbers of lineage-labelled alpha-cell derived cells co-expressing insulin (Fig. 5).

## Discussion

An improved understanding of the adaptive alterations in pancreatic islet cell lineage and identity in ageing should provide insight into the observed progressive loss of glucose homeostasis with age [20]. To this end, we have employed 52-week-old transgenic mice with pancreatic islet beta-cell tracing technology, and directly compared aspects of islet morphology, islet cell turnover rates and related plasticity with 12-week-old control mice. Importantly, at this advanced age, *Ins1*^*Cre/*+^*;Rosa26-eYFP* transgenic mice presented with clear elevations of circulating glucose concentrations, representative of classic age-induced impaired glucose tolerance [21]. In keeping with this, circulating plasma insulin concentrations were equivalent in both groups of mice, despite significantly decreased pancreatic islet and beta-cell areas in the older cohort.

Furthermore, pancreatic islets from older *Ins1*^*Cre/*+^*;Rosa26-eYFP* mice were reduced in number, displaying reductions in total and beta-cell area, partly attributable to decreased beta-cell proliferation and increased beta-cell apoptosis when compared to younger control mice, consistent with age-linked effects described in the human setting [22]. This being despite a significant increase of body weight in the 52-week-old mice, which would generally be considered to augment beta-cell mass [23]. Given that beta-cells can originate from either an endocrine or ductal source [24], the possibility that pancreatic ductal cells could begin to express insulin was also investigated in the two cohorts of mice. Clearly, this process occurs at significant rates in young mice, likely acting as a source of progenitors to help maintain the normal population of functional adult beta-cells [25]. This pathway of beta-cell neogenesis was markedly impaired in older *Ins1*^*Cre/*+^*;Rosa26-eYFP* mice, which may contribute to reduced insulin-positive cell area, although exact causal relationships cannot be established from the present data. Indeed, the static nature of the current analysis does not permit for direct inference of dynamic lineage conversion. Accumulating evidence indicates that ageing tissues exhibit reduced chromatin accessibility and diminished competence of progenitor-like systems [26]. Such age-associated alterations in chromatin state and transcription factor networks may contribute to the restricted ductal plasticity observed. However, the *Ins1*^*Cre/*+^*;Rosa26-eYFP* transgenic mouse model was employed chiefly to study islet and beta-cell endocrine cell fate, meaning the primary focus of our investigations centered around islet cell plasticity in ageing.

In this regard, the number of islet cells positive for both insulin and glucagon was elevated by over 50% in 52 week old *Ins1*^*Cre/*+^*;Rosa26-eYFP* mice when compared to 12 week old controls. Indeed, the appearance of bi-hormonal islet cells has been reported in human T2DM [27], consistent with altered endocrine marker expression patterns that may reflect changes in cellular phenotype within islets [28]. Although, assessment of the islet cell transcription profiles is needed to strengthen this viewpoint. Accordingly, in addition to restricted beta-cell proliferation and increased beta-cell apoptosis, loss of beta-cell identity was heightened in 52-week-old *Ins1*^*Cre/*+^*;Rosa26-eYFP* mice. That said, since molecular cell identity markers were not assessed in the current setting, such interpretations are limited to more descriptive islet cell lineage-labelling patterns. Accordingly, increased expression of neurogenin-3 is linked to beta-cells migrating towards a progenitor endocrine cell state in older mice [29], but single-cell RNA sequencing experiments would be needed to confirm this, that is outside the scope of the current study. Whilst our transgenic mouse models unquestionably enable identification of lineage-labelled islet cell alterations, lack of molecular or dynamic analysis makes definitive conclusions regarding functional identity or lineage transition difficult to confirm. Without molecular profiling or dynamic lineage-tracing analyses, these findings should be interpreted strictly as alterations in lineage-labelled hormone expression rather than definitive evidence of changes in cell identity. Still, observed alterations in hormone marker expression are consistent with previously described changes in beta-cell phenotype during ageing. Several mechanisms have previously been proposed in terms of altered beta-cell phenotype during ageing [30], but such molecular drivers were not examined in the present work. For example, declines in beta-cell identity factors such as Pdx1, MafA and FoxO1 is reported in ageing beta-cells and consistent with the appearance of progenitor-like or bi-hormonal islet cell intermediates [10]. In the current setting, increased loss of insulin expression in original GFP labelled islet cells in ageing *Ins1*^*Cre/*+^*;Rosa26-eYFP* mice, was accompanied by promotion of glucagon and GFP co-positive cell numbers. This represents a discernible shift in cellular hormone expression profiles, in keeping with increased numbers of dual hormone positive islet cells [27]. This beta- to alpha-cell shift is consistent with other models of age- or stress-induced endocrine instability [31]. Moreover, an additional layer of complexity in ageing involves beta-cell senescence [32], that may contribute to both reduced plasticity and less predictable islet cell lineage commitments.

Observations to further probe this in *Glu*^*CreERT2*^;*ROSA26-eYFP* transgenic mice confirmed that islet endocrine cells are less likely to act as a source of new beta-cells in old mice, as compared to their younger counterparts. It is interesting that alpha-cell proliferation was augmented in older *Ins1*^*Cre/*+^*;Rosa26-eYFP* mice, the functional implications of which remain unclear. But this might represent a mechanism to increase the pool of beta-cell progenitor type endocrine cells as also suggested in humans [33], although that hypothesis requires further confirmation. Overall, these findings are consistent with altered endocrine cell phenotype and lineage marker expression in ageing islets. In this regard, it would be particularly interesting to examine the secretory function of any lineage altered islet cells, but methodological restraints mean that this is currently not possible.

Despite the obvious deterioration of pancreatic islet morphology in older mice through the processes outlined above, and these mice presenting with elevated circulating glucose, there are reports of improved beta-cell secretory capacity in aged rodents [34]. This does not translate well to the human setting [35], but aligns with unaltered circulating insulin in the face of reduced functional beta-cell area in 52 week old mice. Thus, although outside the scope of the current study, it would be of interest to investigate glucose responsiveness of islets from 52 week old *Ins1*^*Cre/*+^*;Rosa26-eYFP* mice, alongside assessment of peripheral insulin action using hyperinsulinaemic-euglycemic clamps [36]. In addition to this, age-associated sex differences in glucose homeostasis and metabolism have previously been described [37], and it would be interesting to study the impact of both age and sex on islet cell plasticity in *Ins1*^*Cre/*+^*;Rosa26-eYFP* mice, especially since subtle differences in pancreatic islet morphology have been documented in *Glu*^*CreERT2*^*;ROSA26-eYFP* transgenic mice [38]. Further to this, despite the authenticity of both our transgenic mouse models for assessing islet cell lineage alterations [15–17], alongside robustness of our associated immunohistochemical methods [11, 12, 37, 39], all observations were made at one only time point. As such, a more dynamic evaluation of modifications of islet cell lineage over time would have added to overall interpretation.

In conclusion, ageing in the current transgenic mouse models was associated with marked alterations in pancreatic islet morphology, endocrine cell turnover and patterns of lineage-labelling and hormone marker expression. Older mice exhibited reduced islet number and size, diminished beta-cell proliferation and survival, increased alpha-cell proliferation, and a higher proportion of lineage-labelled cells displaying altered hormone expression profiles. Such alterations of pancreatic islet morphology occurred together with decreased endocrine and ductal cell contributions to insulin-positive islet cell numbers in ageing. Notably however, findings are based on static immunohistochemical analysis and therefore do not establish underlying molecular mechanisms. Whether pharmacological intervention could be used to modulate such pancreatic islets adaptations in ageing remains to be elucidated, but it is encouraging that both clinically approved and experimental drugs for diabetes exert benefits on islet cell plasticity [10, 12, 16, 39]. Future studies incorporating molecular and functional analyses will be required to determine whether the observed cellular changes in ageing have therapeutic implications. The present findings therefore provide descriptive evidence of age-associated remodelling of endocrine cell marker expression, without establishing the underlying molecular mechanisms.

## Supplementary Information

Below is the link to the electronic supplementary material.

Supplementary Material 1

## Data Availability

All data used to support the findings of this study are available from the lead scientist (VD) upon request.
