# Supplementary material for: Adverse effect of age on pancreatic islet morphology, islet cell turnover and lineage-labelled hormone expression in mice
Source: J Endocrinol Invest. 2026 Apr 16;49(8):2045–53. doi: 10.1007/s40618-026-02884-6 (PMC13486126; doi:10.1007/s40618-026-02884-6)
Supplement: Supplementary file 1 [file 40618_2026_2884_MOESM1_ESM.docx]

**Supplementary Table 1:** Target, species, dilution and source of primary and secondary antibodies

| Primary Antibodies | | | |
| --- | --- | --- | --- |
| Target | **Species** | **Dilution** | **Source** |
| Insulin | Mouse | 1/400 | Abcam; ab6995 |
| Glucagon | Rabbit | 1/1000 | Abcam; ab92517 |
| Glucagon | Guinea pig | 1/400 | Raised in-house |
| Ki-67 | Rabbit | 1/500 | Abcam; ab15580 |
| GFP | Goat | 1/500 | Abcam; ab5450 |
| CK-19 | Rabbit | 1/500 | Abcam; ab76539 |
| Secondary Antibodies | | | |
| Target species | **Host Species** | **Dilution** | **Source** |
| Mouse | Donkey | 1/400 | Invitrogen; A-21203 |
| Rabbit | Donkey | 1/400 | Invitrogen; A-21206 |
| Guinea pig | Goat | 1/400 | Invitrogen; A-11073 |
| Goat | Donkey | 1/400 | Invitrogen; A-11055 |
